# The effect of virtual reality glasses used during chest tube removal following open-heart surgery on pain and hemodynamic parameters: a randomized controlled trial

**DOI:** 10.1186/s12872-026-06778-6

**Published:** 2026-10-08

**Authors:** Tuğba Çam Yanik, Muaz Gülşen, Gülay Altun Uğraş, Sevban Arslan, Firdevs Ebru Özdemi̇r

**Affiliations:** 1 Faculty of Nursing, Surgical Nursing Department, Mersin University, Mersin , Türkiye; 2 Faculty of Health Sciences, Surgical Nursing Department, Cukurova University, Adana , Türkiye; 3 Faculty of Nursing, Surgical Nursing Department, Professor, Mersin University, Mersin , Türkiye; 4 Faculty of Health Sciences, Surgical Nursing Department, Professor, Cukurova University, Adana, Türkiye; 5 Cardiovascular Surgery Intensive Care Unit, Mersin University Hospital,, Mersin, Türkiye

**Keywords:** Open-heart surgery, Chest tube, Virtual reality, Pain, Hemodynamic parameters, Nursing

## Abstract

**Background:**

Chest tube removal (CTR) following open-heart surgery is a painful procedure that may adversely affect patients’ hemodynamic parameters.

**Purpose:**

This study aimed to determine the effect of virtual reality glasses (VRG) used during chest tube removal after open-heart surgery on pain levels and hemodynamic parameters.

**Methods:**

This prospective, two-arm (1:1), randomized controlled trial included 82 patients undergoing open-heart surgery. Patients in the VRG group (*n* = 41) were exposed to relaxing forest-walk-themed virtual reality content starting five minutes before CTR and continuing for five minutes after the procedure. The control group (*n* = 41) received routine care without any additional intervention. Pain levels were assessed using the Visual Analog Scale (VAS), and hemodynamic parameters—systolic blood pressure (SBP), diastolic blood pressure (DBP), heart rate (HR), respiratory rate (RR), and peripheral oxygen saturation (SpO₂)—were recorded before CTR, immediately after, and at 5, 15, and 30 min post-procedure.

**Results:**

A significant group × time interaction was observed for pain (*p* = 0.003). After adjustment for baseline pain and multiple comparisons, pain was significantly lower in the VRG group than in the control group immediately after CTR (adjusted mean difference [VRG − Control]: −1.26, 95% CI: −1.85 to − 0.68; adjusted *p* < 0.001), whereas no significant between-group differences were observed at 5, 15, or 30 min. A significant group × time interaction was also observed for SpO₂ (*p* < 0.001), with significantly higher SpO₂ in the VRG group immediately after CTR (adjusted mean difference: 2.70, 95% CI: 1.88 to 3.53; adjusted *p* < 0.001). No significant group × time interactions were observed for SBP, DBP, HR, or RR.

**Conclusion:**

VRG use during CTR was associated with lower pain immediately after the procedure and higher SpO₂ immediately after CTR. No significant effects were observed for SBP, DBP, HR, or RR. These findings suggest that VRG may be a useful non-pharmacological adjunct for short-term pain management during CTR following open-heart surgery. Further studies are needed to confirm its effects on oxygenation and other hemodynamic parameters.

**Trial registration:**

ClinicalTrials.gov (NCT05585853) first submitted on October 12, 2022, and first posted on October 19, 2022.

**Supplementary Information:**

The online version contains supplementary material available at https://doi.org/10.1186/s12872-026-06778-6.

## Introduction

Open-heart surgery is a major surgical procedure widely used in the treatment of cardiovascular diseases. Because this procedure requires advanced techniques, such as extensive tissue dissection and cardiopulmonary bypass, the recovery process can be prolonged and challenging [1, 2]. Postoperatively, pain associated with incision sites, as well as pain caused by invasive chest tubes (CTs) placed to prevent complications, represents a significant clinical concern. In particular, the pain experienced during the removal of chest tubes can lead to haemodynamic instability in patients [1, 3, 4].

The primary purpose of using a chest tube (CT) in patients following open-heart surgery is to ensure the drainage of blood, air, and fluid accumulated in the chest cavity [1, 3, 5]. CTs are typically removed when drainage is less than 100 mL within the previous 24 h, the lungs are fully expanded, and no air leak is present [2, 3]. Although CTs help reduce the risk of complications and accelerate postoperative recovery, their adhesion to surrounding tissues after placement can cause acute and severe pain. Consequently, the routine removal of CTs is a painful procedure that may trigger significant stress responses in patients [2, 3, 6, 7]. In individuals with high cardiovascular sensitivity, such as those who have undergone cardiac surgery, pain can initiate a neuroendocrine response that negatively affects haemodynamic parameters, including heart rate (HR), systolic blood pressure (SBP), diastolic blood pressure (DBP), and respiratory rate (RR) [8]. Therefore, effective pain management prior to chest tube removal (CTR) following cardiac surgery is of critical importance for enhancing patient comfort and reducing the risk of complications [4, 8, 9].

During CTR, mediators such as bradykinin, kinins, and histamine are released as part of the inflammatory response, lowering the pain transmission threshold. This process can lead to peripheral sensitization and hyperalgesia, resulting in increased pain sensitivity beyond normal tolerance and a heightened perception of pain [7, 10, 11]. In addition to the direct effects of pain, sympathetic nervous system activity increases, leading to elevations in HR, SBP, DBP, and RR, while peripheral oxygen saturation (SpO₂) may decrease [1, 8, 9]. Furthermore, patients’ fear of experiencing pain prior to the procedure can exacerbate pain levels through nociceptive responses mediated by the limbic system. Therefore, identifying effective strategies to manage pain associated with CTR during the postoperative period is essential for reducing cardiac workload and improving long-term outcomes [3, 10, 12].

Non-pharmacological methods are increasingly used for pain management during CTR following cardiac surgery [1, 8], including virtual reality glasses (VRG) [1, 2, 9, 13]. VRG provides an interactive audiovisual environment that may reduce pain perception through distraction [1, 5]. Several individual studies have reported reductions in pain associated with VRG use [5, 9, 13, 14]; however, the overall evidence remains inconsistent. A systematic review and meta-analysis by Micheluzzi et al. found no statistically significant effect of VR on pain compared with standard care [15]. Previous studies have reported lower pain levels during and after CTR with VRG use [1, 2], while another study reported differences in SBP, DBP, HR, and RR following CTR [9].

Although previous studies have investigated the effects of VRG on pain during CTR following open-heart surgery [1, 2, 9], evidence regarding its effects on pain and physiological parameters assessed at multiple time points remains limited. Therefore, this study aimed to determine the effect of VRG used during CTR following open-heart surgery on pain and physiological parameters. We hypothesized that, compared with routine care, VRG use during CTR would reduce pain intensity and favorably affect physiological parameters, including systolic blood pressure, diastolic blood pressure, heart rate, respiratory rate, and SpO₂.

## Method

### Aim and study design

**Fig. 1:**
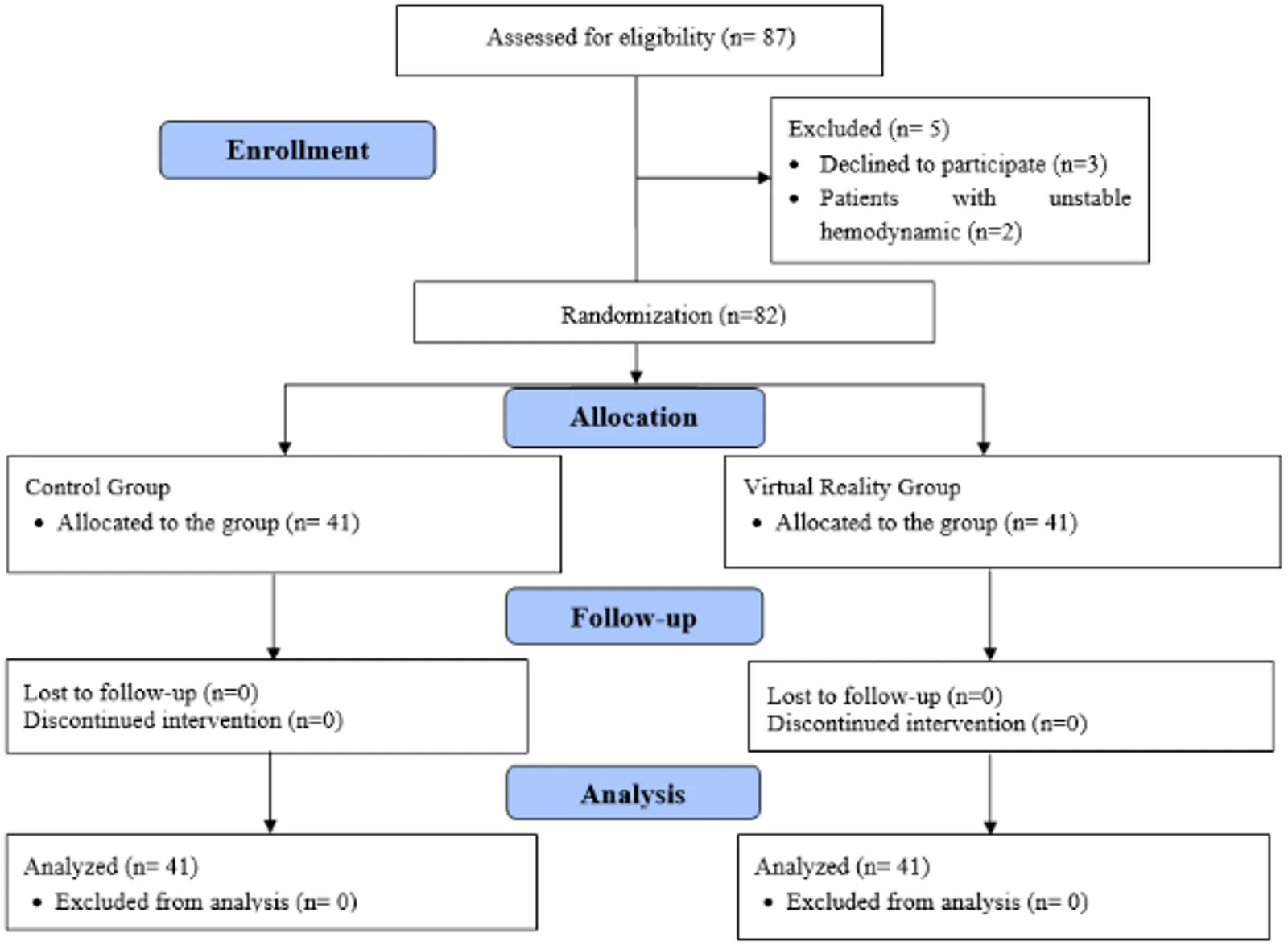
CONSORT flow diagram

This prospective, parallel-group, two-arm (1:1) randomized controlled trial aimed to evaluate the effect of virtual reality glasses (VRG) applied during chest tube removal (CTR) following open-heart surgery on postoperative pain and hemodynamic parameters (SBP, DBP, HR, RR, and SpO₂). This randomized controlled trial was reported in accordance with the Consolidated Standards of Reporting Trials (CONSORT) guidelines [16]. The study was registered with ClinicalTrials.gov (Identifier: NCT05585853) and completed without protocol modifications. The study protocol was prospectively registered with ClinicalTrials.gov (NCT05585853) in 2022. The ethical approval process took longer than initially anticipated and was completed in July 2023. Following ethical approval, initiation of participant recruitment was delayed due to clinical scheduling considerations and the availability of eligible patients undergoing open-heart surgery. Participant recruitment therefore commenced in January 2025. No protocol modifications were made during the study. The CONSORT flow diagram is presented in Fig. 1.

### Primary and secondary objectives

The primary objective of this study was to determine the effect of VRG use during chest tube removal on pain intensity in patients undergoing open-heart surgery. The secondary objective was to determine the effect of VRG use on physiological parameters, including systolic blood pressure (SBP), diastolic blood pressure (DBP), heart rate (HR), respiratory rate (RR), and peripheral oxygen saturation (SpO₂).

### Research hypotheses

#### H₁

There is a difference between the VRG group and the control group in terms of pain levels experienced during CTR.

#### H_2_

There is a difference between the VRG group and the control group in terms of hemodynamic parameters (SBP, DBP, HR, RR, SpO₂) during CTR.

### Population and sampling

The study sample consisted of 82 patients undergoing open-heart surgery who were hospitalized in the Cardiovascular Surgery Intensive Care Unit of a university hospital in Mersin, Turkey, between January 1, 2025, and August 26, 2025.

An a priori power analysis was conducted to determine the required sample size. The study by Guo et al. (2015) was used as a reference [17]. In that study, the mean Visual Analog Scale (VAS) score following VRG was 2.63 ± 1.27 in the intervention group and 7.64 ± 3.41 in the control group, corresponding to a large standardized effect. To avoid basing the sample-size calculation on this very large observed effect, a more conservative effect size of Cohen’s d = 0.80 was used in the present study [18]. With a two-sided significance level of 0.05 and 90% statistical power, the minimum required sample size was calculated as 68 participants (34 per group) [19]. To allow for potential participant loss and to increase the robustness of the study, the minimum sample size was increased by approximately 20% (68 × 1.20 = 81.6), resulting in a target sample size of 82 participants (41 per group). Sample-size calculations were performed using G*Power software [20].

#### Inclusion criteria

The inclusion criteria were as follows: (1) age ≥ 18 years; (2) undergoing elective open-heart surgery; (3) being scheduled for chest tube removal on the second postoperative day; (4) being hemodynamically stable; (5) being alert and cooperative and having sufficient cognitive capacity to understand and follow the study and VRG instructions; (6) being able to communicate in Turkish; (7) having no previous experience of chest tube removal; and (8) providing written informed consent to participate in the study.

#### Exclusion criteria

The exclusion criteria were as follows: (1) hemodynamic instability; (2) a visual impairment that prevented adequate viewing of or engagement with the VR content; (3) sensitivity, injury, or trauma that prevented the appropriate use of the VRG; and (4) unwillingness to participate in or continue the study.

During the study, 87 patients underwent open-heart surgery. Of these, 3 patients declined participation and 2 had an unstable hemodynamic status. Therefore, 5 patients were excluded, and 82 patients were included in the study (Fig. 1).

### Randomization

Block randomization was used to allocate the 82 patients to two groups in a 1:1 ratio. The randomization sequence was computer-generated by an independent statistician who was not involved in participant recruitment, intervention delivery, or data collection. Before participant recruitment, a random draw was used to designate Group A as the control group and Group B as the VRG group. Group assignments were placed in sequentially numbered, opaque, sealed envelopes and kept by the coordinating investigator (G.A.U.). After admission to the intensive care unit (ICU) following open-heart surgery, patients were assessed for eligibility by the researcher (F.E.Ö.). After eligibility was confirmed and written informed consent was obtained, the next sequentially numbered envelope was used to determine group allocation. Thus, the allocation sequence remained concealed until participant enrollment and assignment. The VRG intervention was administered by a single researcher (F.E.Ö.) according to a standardized protocol.

The study included patients undergoing open-heart surgery who were scheduled for CTR on the second postoperative day, were able to speak and understand Turkish, had stable hemodynamic parameters, were aged ≥ 18 years, were alert and cooperative, had undergone elective surgery, had no prior CT experience, had no sensitivity or trauma in the VRG application area, and voluntarily agreed to participate. During the study, 87 patients underwent open-heart surgery. Of these, patients who declined participation (*n* = 3), had unstable hemodynamic status (*n* = 2), or did not meet the inclusion criteria were excluded. A total of 5 patients were excluded (Fig. 1).

### Blinding

Due to the nature of the intervention, participants and the researcher who administered the VRG intervention and collected the outcome data (F.E.Ö.) could not be blinded to group allocation. After completion of data collection, the dataset was coded using group labels (A and B) by an independent researcher (T.Ç.Y.). Statistical analyses were subsequently performed by an independent statistician who was blinded to the identity of the treatment groups.

### Data collection tools

The “Demographic Information Form,” “Visual Analog Scale,” and “Hemodynamic Parameters Monitoring Form” were used for data collection.

#### Descriptive characteristics form

The Descriptive Characteristics Form was developed in line with the study aims and hypotheses, based on relevant literature [8, 13, 21, 22]. It consists of six items assessing patients’ sociodemographic characteristics (age, gender, marital status, educational level) and surgical characteristics (diagnosis and type of surgery).

#### Visual analog scale (VAS)

The VAS is a reliable and widely used tool consisting of a 10-cm line with endpoints labeled “no pain” (0 cm) and “maximum pain” (10 cm). Participants mark a point on the line corresponding to their pain level, which is then measured in centimeters to obtain a numerical value [23, 24].

#### Hemodynamic parameters monitoring form

This form records SBP, DBP, HR, RR, and SpO₂ values in both groups at the following time points: 5 min before CTR, immediately after CTR, and at 5, 15, and 30 min after CTR.

### Data collection

As part of routine postoperative care, all patients received tramadol (100 mg) by intravenous infusion. The infusion was initiated approximately 30 min before awakening and continued at a rate of 5 mg/h until discharge from the intensive care unit. No additional analgesic medication was administered specifically for chest tube removal in either group. To standardize the procedure and minimize operator-related variability, all chest tubes were removed by the same physician using the same routine clinical procedure.

Before CTR, all patients completed the “Descriptive Characteristics Form,” VAS, and the Hemodynamic Parameters Monitoring Form. Five minutes before CTR, patients in the VRG group were fitted with a virtual reality device (VR Box Virtual Reality Headset 3D VR Glasses V2.0, 2020 model) connected to an Android smartphone (Lenovo P2a42) while in the semi-Fowler position. Patients viewed relaxing forest-walk-themed videos with an average duration of 10 min before, during, and after CTR. CTR was performed routinely by a physician five minutes after the VRG intervention began. During the procedure, patients were positioned in the semi-Fowler position and instructed to take a deep breath and hold it while the chest tube was removed. After CTR, the VRG intervention continued for an additional five minutes. Pain levels and hemodynamic parameters were assessed 5 min before CTR (coinciding with the start of the VR intervention), immediately after CTR, 5 min after CTR (coinciding with the end of the VR intervention), and at 15 and 30 min after CTR.

The control group received routine care, and measurements were taken at the same time intervals. Hemodynamic parameters were monitored using a bedside monitor (Vismo, Nihon Kohden, Germany), while RR was measured manually by observing chest movements for one minute. All patients received routine care, including 100 mg tramadol infusion for pain management. No additional analgesics were administered within approximately 2 h before or after CTR. No patient reported discomfort during VRG, and no procedures were discontinued.

### Data analysis

Descriptive statistics were presented as frequencies, percentages, means, standard deviations, and medians (min–max). Categorical variables were analyzed using the Pearson χ² test or Fisher’s exact test, as appropriate. The normality of continuous variables was assessed using the Shapiro–Wilk test. For between-group comparisons of continuous variables, the independent-samples t-test was used for normally distributed data, whereas the Mann–Whitney U test was used for non-normally distributed data. Repeated measurements of pain and hemodynamic parameters were analyzed using linear mixed-effects models with a participant-specific random intercept to account for within-participant correlation. For each outcome, post-CTR measurements were modeled as the dependent variable, with group, time, and the group × time interaction included as fixed effects. The corresponding baseline value was included as a covariate, and time was treated as a categorical variable. When a statistically significant group × time interaction was identified, post-hoc between-group contrasts at individual post-CTR time points were performed with Bonferroni adjustment for multiple comparisons. Treatment effects are presented as adjusted mean differences with 95% confidence intervals (CIs). All tests were two-sided, and statistical significance was set at *p* < 0.05.

## Results

Patients in the control and VRG groups were similar in terms of baseline characteristics (*p* > 0.05; Table 1).

**Table 1:** Comparison of the demographic characteristics of patients (*n* = 82)

Characteristics	Control Group Mean ± SD		Virtual Reality Group Mean ± SD		Test value	*p*
**Age**, years* (range: 37–82)	61.83 ± 9.34		61.24 ± 10.95		1.991	0.795
Gender **	**n**	**%**	**n**	**%**		
Female	11	26.8	6	14.6	1.855	0.173
Male	30	73.2	35	85.4		
Marital status**						
Single	2	4.9	1	2.4	1.000	-
Married	39	95.1	40		97.6	
**Education level** ^**€**^						
Illiterate	4	9.8	-	-	820	0.560
Literate	-	-	22	53.7		
Primary school	25	61	10	24.4		
High school	11	26.8	9	22		
University	1	2.4	-	-		
Diagnosis**						
CAD	34	82.9	36	87.8	0.390	0.532
HF	7	17.1	5	12.2		
Current surgical intervention**						
CABG	34	82.9	36	87.8	0.390	0.532
VR	7	17.1	5	12.2		
Previous history of surgery**						
Yes	17	41.5	14	34.1	3.170	0.075
*1*	*11*	26.8	9	22.1		
*2*	*4*	9.8	5	12		
*3*	*2*	4.9	-	-		
No	24	58.5	27	65.9		

CAD: Coronary Artery Disease; HF: Heart Failure; CABG: Coronary Artery Bypass Graft; VR: Valve Replacement

*A t-test was used for data analysis in independent groups. **A chi-square test was used for data analysis. [Pearson Chi-Square: gender, diagnosis, type, history of prior AMI; Fisher’s Exact Test: marital status] ^€^The Mann-Whitney U test was used for independent groups

### Pain Levels

Linear mixed-effects model analysis showed a significant group × time interaction for pain (*p* = 0.003). After adjustment for baseline pain and multiple comparisons, pain was significantly lower in the VRG group than in the control group immediately after CTR (adjusted mean difference [VRG − Control]: −1.26, 95% CI: −1.85 to − 0.68; adjusted *p* < 0.001). No significant between-group differences were observed at 5, 15, or 30 min after CTR following Bonferroni adjustment (Table 2).

### Hemodynamic parameters

Linear mixed-effects model analysis showed a significant group × time interaction for SpO₂ (p < 0.001). Immediately after CTR, SpO₂ was significantly higher in the VRG group than in the control group (adjusted mean difference [VRG − Control]: 2.70, 95% CI: 1.88 to 3.53; adjusted p < 0.001). Following Bonferroni adjustment, no significant between-group differences in SpO₂ were observed at 5, 15, or 30 min after CTR. No significant group × time interactions were observed for SBP, DBP, HR, or RR (all p > 0.05) (Table 2).

**Table 2:** Comparison of pain and hemodynamic parameters between the groups (*n* = 82)

Pain (VAS)	Assessment Time	Control Group Mean ± SD	VR Group Mean ± SD	Adjusted mean difference, VR − Control (95% CI)	Adjusted p*	Group × Time *p*
						0.003
	Before CTR^a^	3.76 ± 2.49	2.61 ± 1.51	-	-	
	Immediately after CTR^b^	6.80 ± 2.34	5.12 ± 1.58	**−1.26 (− 1.85 to − 0.68)**	**< 0.001**	
	5 min after CTR^c^	4.24 ± 1.85	3.41 ± 1.28	−0.41 (− 0.99 to 0.18)	0.692	
	15 min after CTR^d^	2.66 ± 1.57	1.95 ± 0.97	−0.28 (− 0.87 to 0.30)	1.000	
	30 min after CTR^e^	2.19 ± 1.38	1.59 ± 0.74	−0.19 (− 0.77 to 0.40)	1.000	
**SBP (mmHg)**						0.847
	Before CTR^a^	121.66 ± 22.61	132.15 ± 21.00	-	-	
	Immediately after CTR^b^	129.93 ± 25.02	138.07 ± 19.22	0.87 (− 4.53 to 6.28)	-	
	5 min after CTR^c^	126.24 ± 20.83	133.80 ± 17.41	0.29 (− 5.12 to 5.70)	-	
	15 min after CTR^d^	121.17 ± 20.14	127.32 ± 17.41	−1.13 (− 6.53 to 4.28)	-	
	30 min after CTR^e^	119.88 ± 19.10	127.93 ± 16.01	0.78 (− 4.63 to 6.19)	-	
**DBP (mmHg)**						0.656
	Before CTR^a^	60.71 ± 13.08	62.49 ± 12.45	-	-	
	Immediately after CTR^b^	63.90 ± 14.73	66.07 ± 13.14	0.70 (− 2.17 to 3.58)	-	
	5 min after CTR^c^	60.98 ± 12.15	65.10 ± 11.87	2.66 (− 0.22 to 5.53)	-	
	15 min after CTR^d^	58.49 ± 11.38	62.31 ± 11.99	2.36 (− 0.52 to 5.24)	-	
	30 min after CTR^e^	58.54 ± 11.58	61.95 ± 12.70	1.95 (− 0.93 to 4.83)	-	
**HR (beats/min)**						0.330
	Before CTR^a^	96.15 ± 14.49	94.95 ± 14.00	-	-	
	Immediately after CTR^b^	96.41 ± 15.52	98.85 ± 15.77	3.52 (0.77 to 6.27)	-	
	5 min after CTR^c^	93.63 ± 13.76	94.76 ± 14.66	2.20 (− 0.55 to 4.95)	-	
	15 min after CTR^d^	91.83 ± 13.00	92.85 ± 14.11	2.10 (− 0.65 to 4.85)	-	
	30 min after CTR^e^	92.51 ± 15.45	92.54 ± 12.14	1.10 (− 1.65 to 3.85)	-	
**RR (breaths/min)**						0.587
	Before CTR^a^	24.12 ± 3.98	22.90 ± 4.37	-	-	
	Immediately after CTR^b^	25.63 ± 4.40	25.56 ± 3.46	0.65 (− 0.64 to 1.94)	-	
	5 min after CTR^c^	25.10 ± 4.21	24.51 ± 3.70	0.14 (− 1.15 to 1.42)	-	
	15 min after CTR^d^	23.85 ± 4.22	23.00 ± 3.61	−0.13 (− 1.42 to 1.16)	-	
	30 min after CTR^e^	23.07 ± 3.92	22.68 ± 3.36	0.33 (− 0.96 to 1.62)	-	
**SpO₂ (%)**						<0.001
	Before CTR^a^	94.17 ± 3.97	93.02 ± 3.56	-	-	
	Immediately after CTR^b^	91.54 ± 3.81	93.24 ± 3.58	**2.70 (1.88 to 3.53)**	**< 0.001**	
	5 min after CTR^c^	93.02 ± 4.41	92.90 ± 3.55	0.87 (0.05 to 1.70)	0.152	
	15 min after CTR^d^	93.44 ± 4.01	93.32 ± 3.48	0.87 (0.05 to 1.70)	0.152	
	30 min after CTRe	93.66±4.01	93.63±3.44	0.97 (0.15 to 1.80)	0.084	

Values are presented as mean ± standard deviation (SD). Repeated post-CTR measurements were analyzed using linear mixed-effects models with a participant-specific random intercept. Group, time, and group × time interaction were included as fixed effects, and the corresponding baseline value was included as a covariate. Adjusted mean differences represent the estimated difference between the VRG and control groups (VRG − Control) with 95% confidence intervals (CI). Bonferroni-adjusted p values are reported for post-hoc between-group comparisons when the group × time interaction was statistically significant. Statistical significance was set at *p* < 0.05. CTR: chest tube removal; VR: virtual reality; SBP: systolic blood pressure; DBP: diastolic blood pressure; HR: heart rate; RR: respiratory rate; SpO₂: peripheral oxygen saturation; CI: confidence interval

*The immediate post-CTR assessment was performed immediately after complete removal of the chest tube

## Discussion

The findings of this study demonstrated a significant group × time interaction for pain, with significantly lower pain in the VRG group immediately after CTR after adjustment for baseline pain and multiple comparisons. However, this between-group difference was not maintained at 5, 15, or 30 min after CTR. A significant group × time interaction was also observed for SpO₂, with higher SpO₂ in the VRG group immediately after CTR, whereas no significant group × time interactions were observed for SBP, DBP, HR, or RR. These findings suggest that the effects of VRG were primarily limited to immediate post-procedural pain and oxygenation.

### The effect of VRG on pain

Due to the dense innervation of the chest wall and pleural surface, CTR is known to cause severe acute pain [3, 11, 25]. A meta-analysis by Tsai et al. reported that non-pharmacological interventions may be beneficial for pain management during CTR [11]. Different non-pharmacological interventions may reduce pain through intervention-specific mechanisms. For VR-based interventions, the analgesic effect has primarily been attributed to attentional distraction and modulation of pain perception, whereas mechanisms such as reduced nerve conduction velocity are more specifically associated with interventions such as cold application [3, 6, 11, 12, 26].

Consistent with earlier studies involving patients undergoing open-heart surgery [1, 2, 9], the present study found that pain was significantly lower in the VRG group than in the control group immediately after CTR. However, although the mean pain score remained numerically lower in the VRG group at 5 min after CTR, the adjusted between-group difference was not statistically significant. Therefore, the analgesic effect observed in this study appears to have been limited to the immediate post-procedural period. Özbaş et al. (2024) reported that VRG significantly reduced pain during CTR and up to 10 min afterward [9]. Similarly, Seçer et al. (2025) demonstrated that VRG was effective in reducing pain up to 10 min post-CTR in post-CABG patients [2], while Dalir et al. found sustained effects up to 15 min [1]. In the present study, however, the significant between-group difference was confined to the immediate post-CTR assessment and was not maintained at 5, 15, or 30 min after adjustment for baseline pain and multiple comparisons. Differences in VR content, duration and degree of immersion, as well as individual demographic and psychosocial characteristics, may contribute to differences in the duration of the observed effects across studies. Previous literature also supports the effectiveness of VRG in reducing pain in other surgical populations [13, 27]. For example, Pandgrai et al. (2022) reported reduced pain immediately after head and neck surgery with VRG use [27], and Gökçe and Arslan demonstrated reduced pain during and immediately after catheter removal following coronary angiography [13].

In the present study, the 10-minute VRG application, beginning 5 min before CTR and continuing for 5 min after the procedure, may have reduced attention to the painful stimulus by providing an immersive three-dimensional experience. VR-based analgesia has been proposed to act primarily through attentional distraction and modulation of pain perception [1, 2, 9, 12, 13]. However, because autonomic nervous system activity was not directly measured in this study, an autonomic mechanism cannot be established from the present findings. Overall, the results suggest that VRG may be useful as a non-pharmacological adjunct for reducing immediate procedural pain associated with CTR, although the analgesic effect was not sustained at subsequent assessment time points.

CTR is a highly painful procedure that can activate the sympathetic nervous system, leading to increases in SBP, DBP, HR, and RR [11, 12]. Previous studies have reported varying effects of VRG on these parameters. Özbaş et al. reported reductions in SBP, DBP, HR, and RR associated with VRG use [9], while studies conducted in other procedural and surgical settings have also reported changes in cardiovascular parameters following VR-based interventions [27].

### The effect of VRG on hemodynamic parameters

CTR is a highly painful procedure that can activate the sympathetic nervous system, leading to increases in SBP, DBP, HR, and RR [11, 12]. Previous studies have reported varying effects of VRG on these parameters. Özbaş et al. reported reductions in SBP, DBP, HR, and RR associated with VRG use [9], while studies conducted in other procedural and surgical settings have also reported changes in cardiovascular parameters following VR-based interventions [27].

In line with the literature [9, 28, 29], no significant group × time interactions were observed for SBP, DBP, HR, or RR in the present study. Therefore, the findings do not provide evidence of an overall effect of VRG on these physiological parameters over time. However, a significant group × time interaction was observed for SpO₂, and SpO₂ was significantly higher in the VRG group immediately after CTR after adjustment for baseline SpO₂ and multiple comparisons. As the significant between-group difference was observed only immediately after CTR and was not maintained at subsequent assessment time points, this finding should be interpreted cautiously. Previous studies suggest that immersive VR may influence autonomic and stress-related physiological responses; however, the direction and magnitude of these responses may vary depending on the VR content, level of immersion, and clinical context [14, 30, 31]. As autonomic nervous system activity was not directly assessed in the present study, such mechanisms cannot be inferred from our findings.

VR environments may influence parasympathetic activity by diverting attention away from painful stimuli, which may contribute to changes in heart rate and blood pressure [14]. Previous studies have reported favorable effects of VRG on hemodynamic parameters [9, 13, 14]. A notable finding of the present study was that SpO₂ levels were significantly higher in the VRG group than in the control group immediately after CTR after adjustment for baseline SpO₂ and multiple comparisons. İnce and Özlü also reported improvements in SpO₂ following VRG [14], whereas Grahanli et al. found no significant effect [29]. The difference observed in the present study may be related to the use of VRG during a highly painful procedure such as CTR following cardiac surgery. Pain-induced shallow breathing may adversely affect oxygenation [8, 11, 12]. By altering attention and promoting relaxation, VRG may potentially influence breathing patterns and thereby support oxygen saturation levels [32, 33]. However, because respiratory mechanisms and autonomic nervous system activity were not directly assessed in the present study, the mechanism underlying the observed SpO₂ difference cannot be determined.

### Limitations of the study

However, several limitations should be noted. First, the effects of VRG were assessed only up to 30 min after CTR, limiting conclusions regarding its longer-term effectiveness. Second, the absence of a placebo or active comparison group, such as a non-immersive video group, limits the ability to isolate the specific effect of VR immersion. Third, owing to the nature of the intervention, participants and the researcher who administered the VRG intervention and collected the outcome data could not be blinded to group allocation, which may have introduced performance and measurement bias. Fourth, participants’ comorbidities were not systematically assessed or compared between the groups. In particular, pre-existing respiratory conditions may have influenced RR and SpO₂ values and contributed to variability in these physiological outcomes. Therefore, the physiological findings, especially those related to SpO₂, should be interpreted cautiously. Finally, because this study was conducted at a single center, the generalizability of the findings to other institutions and clinical settings may be limited.

## Conclusion

The use of VRG during chest tube removal was associated with lower pain immediately after the procedure. No significant group × time interactions were observed for SBP, DBP, HR, or RR. A significant group × time interaction was observed for SpO₂, with significantly higher SpO₂ in the VRG group immediately after CTR; however, significant between-group differences were not observed at subsequent assessment time points, and this finding should therefore be interpreted cautiously. These findings suggest that VRG may be a useful non-pharmacological adjunct for reducing immediate pain during chest tube removal following open-heart surgery. Further multicenter studies are needed to confirm these findings and to clarify the effects of VRG on hemodynamic outcomes

## Impact statement

### What is known about this topic

- Chest tube removal following open-heart surgery is widely recognized as one of the most painful postoperative procedures and is associated with significant physiological stress responses.
- Pharmacological interventions are commonly used for pain management during this procedure; however, they may cause adverse effects and may not fully relieve patient discomfort.
- Non-pharmacological approaches, particularly distraction-based techniques, are increasingly recommended; however, evidence regarding the effectiveness of virtual reality (VR) in cardiovascular surgery patients remains limited.

### What this paper adds

- This randomized controlled trial showed that pain was significantly lower in the VRG group immediately after chest tube removal in patients undergoing open-heart surgery.
- The study also identified a higher SpO₂ value in the VRG group immediately after chest tube removal; however, this finding was not sustained at subsequent assessment time points and should be interpreted cautiously.
- The findings suggest that VRG may be a feasible non-pharmacological nursing intervention for reducing immediate pain associated with chest tube removal following open-heart surgery.

## Supplementary Information

Below is the link to the electronic supplementary material.

Supplementary Material 1

## Data Availability

The datasets generated and/or analyzed during the current study are available from the corresponding author on reasonable request.
